# Different Methodologies Result in Opposite Patterns of Diversification Rates in Flowering Plants Across Latitudes

**DOI:** 10.1002/ece3.73010

**Published:** 2026-01-28

**Authors:** Hong Qian, Michael Kessler

**Affiliations:** ^1^ Research and Collections Center Illinois State Museum Springfield Illinois USA; ^2^ Department of Systematic and Evolutionary Botany University of Zurich Zurich Switzerland

**Keywords:** angiosperm, latitudinal diversification gradient, speciation, species richness

## Abstract

There are two major groups of methods estimating diversification rates: One group depends on well resolved phylogenies and the other is phylogeny‐free. Previous studies on angiosperms (flowering plants) have used phylogeny‐based methods to estimate diversification rates and concluded that diversification rates increase with increasing latitudes, a trend opposite to that of liverworts, mosses and ferns, for which diversification rates were estimated with the method‐of‐moments estimator, a nonphylogeny method. In this study, we use the method‐of‐moments estimator to estimate diversification rates of angiosperm species within genera worldwide. We find that diversification rates decrease with increasing latitudes. The result of our study is thus contrary to those of previous studies on angiosperms. We discuss the degree to which these differences are due to methodological limitations of using phylogeny‐based approaches to estimate diversification rates of angiosperms. More data from poorly studied angiosperm groups are needed so that we can move toward developing well‐resolved species‐level phylogenies for angiosperms, which will allow a better assessment of the robustness of various diversification estimation methods.

## Graphical abstract

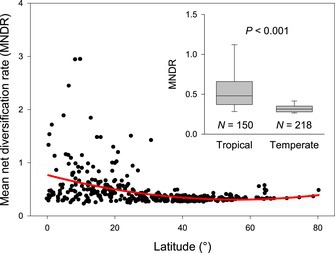

## 1 Introduction

Investigating global variation of diversification rates is a major goal in macroecology and evolutionary biology (Wiens 2015b; Title et al. 2025). Several methodological approaches have been developed to estimate diversification rates (Morlon 2014). They can be roughly divided into two major groups: nonphylogeny‐based approaches versus phylogeny‐based approaches. Among nonphylogeny‐based approaches, the method‐of‐moments estimator (Magallón and Sanderson 2001), also known as the MS approach (Meyer and Wiens 2018), provides a tractable way to estimate net diversification rate by dividing the log‐transformed species richness of a lineage by the age of the lineage (Stanley 1979; Magallón and Sanderson 2001). This method, which does not require full phylogenetic trees, has been used to address many questions related to diversification in different groups of organisms, including both plants such as angiosperms (Eriksson and Bremer 1992; Magallón and Sanderson 2001; Davies et al. 2004; Hughes and Eastwood 2006; Boucher et al. 2020; Wu and Wiens 2022), ferns (Qian et al. 2025), mosses (Qian 2025), liverworts (Maul et al. 2025; Qian et al. 2026), and animals such as ants (Economo et al. 2018), birds (Cooney et al. 2016; Rabosky and Matute 2013), amphibians (Adams et al. 2009; Rabosky and Adams 2012; Gómez‐Rodríguez et al. 2015; Marin and Hedges 2016), and fishes (Alfaro et al. 2007; Tedesco et al. 2017), in addition to those covering multiple phyla of animals (Wiens 2015a, 2015b) or covering both plants and animals (Scholl and Wiens 2016). This approach is particularly useful for studies on organisms for which a species‐level phylogeny is not available.

Phylogeny‐based diversification rate estimators include, but are not limited to, Bayesian analysis of macroevolutionary mixtures (BAMM; Rabosky 2014) and mean root distance (MRD; Tietje et al. 2022). Phylogeny‐based diversification rate estimators have also been used in different groups of animals and plants, including angiosperms (Igea and Tanentzap 2020; Dimitrov et al. 2023; Tietje et al. 2022), gymnosperms (Jin et al. 2021), ferns (Wu et al. 2025), and ants (Economo et al. 2018). The robustness of diversification rates derived from a phylogeny‐based approach heavily depends on the quality of phylogenetic trees on which the diversification rates are estimated.

Several studies have reported latitudinal patterns of diversification rates in angiosperms (flowering plants) at a global scale. For example, using the BAMM approach, Igea and Tanentzap (2020) and Dimitrov et al. (2023) show that speciation or diversification rates in angiosperms are higher towards high latitude. Using the MRD approach, Tietje et al. (2022) also show that diversification rates in angiosperms are higher in temperate regions than in tropical regions. However, the conclusions of these studies, which all used phylogeny‐based diversification rate estimators, are opposite to those for liverworts (Qian et al. 2026), mosses (Qian 2025) and ferns (Qian et al. 2025), for which the method‐of‐moments estimator was used to estimate diversification rates. It thus needs to be tested whether the contradictory results for different groups of plants reflect differences in evolutionary histories among taxa (e.g., seed plants versus non‐seed plants) or differences among the approaches used to estimate diversification rates (e.g., phylogeny‐based versus nonphylogeny‐based approaches).

Dimitrov et al. (2023) used the BAMM approach to estimate diversification rates for species within each genus of angiosperms and explored latitudinal patterns of mean diversification rates in political regions across the world. Here, we use the method‐of‐moments estimator to estimate diversification rates for species within each genus based on genus‐stem ages derived by Dimitrov et al. (2023), and explore latitudinal patterns of mean diversification rates in political regions across the world. Because these two studies use the same data of genus evolutionary histories (divergence times) and use similar data for plant distributions across the world, if the present study finds a trend of decreasing diversification rates towards higher latitudes as in Qian et al. (2026) for liverworts, Qian (2025) for mosses, and Qian et al. (2025) for ferns, then the difference between the present study and Dimitrov et al. (2023) could be attributed to differences in the two groups of diversification rate estimators (i.e., phylogeny‐based versus non‐phylogeny‐based estimators).

## 2 Materials and Methods

The method‐of‐moments estimator is expressed as ln[*n*(1—ε) + ε]/*t*, where *n* is the number of extant species in a lineage (i.e., a genus in our case), *t* is the age of the lineage, and ε is the relative extinction rate suggested to vary from 0 to 0.9 (Magallón and Sanderson 2001). Because using different values of ε has little impact on geographic patterns of diversification rates (Qian 2025; Stephens et al. 2025), we followed previous studies (e.g., Davies et al. 2004; Boucher et al. 2020; Maul et al. 2025; Qian 2025; Qian et al. 2026) in using 0 as the value of ε. We obtained the information on the stem age of each genus from Dimitrov et al. and the number of species in each genus from the World Checklist of Vascular Plants (WCVP) database (https://powo.science.kew.org/about‐wcvp).

We assigned a total of 326,942 (97%) of the angiosperm species in the world to the genera with ages in Dimitrov et al. We obtained a species list of native angiosperms in each of 368 geographic units at Taxonomic Databases Working Group (TDWG) level 3 (described in Brummitt 2001) from WCVP, and calculated mean net diversification rate for each geographic unit. Specifically, for each geographic unit, each of its species was assigned the diversification rate of its genus, and the mean net diversification rate for each geographic unit was calculated as the arithmetic mean net diversification rate across all species in the geographic unit. This is a standard approach for analyses of MDR (e.g., Adams et al. 2009; Hutter et al. 2017; Wu and Wiens 2022; Maul et al. 2025; Qian 2025; Qian et al. 2025, 2026). Because this approach of calculating mean diversification rates is analogous to the one used by Dimitrov et al. and the raw data that we used are largely the same as those used by Dimitrov et al., differences in the results between the two studies can mainly be attributed to differences between the BAMM as used in Dimitrov et al. and MS methods.

To explore variation in diversification rate between tropical and temperate latitudes, we divided the globe into two broad climate zones: tropical zone (latitudes between 23.5°N and 23.5°S) and temperate zone (latitudes south of 23.5°S or north of 23.5°N). A geographic region was assigned to either of the climatic zones according to the centroid of the latitude of the geographic region. Significance in difference of mean diversification rates between tropical and temperate zones was assessed by *t*‐test, using the packages SYSTAT (Wilkinson et al. 1992).

## 3 Results

**FIGURE 1:**
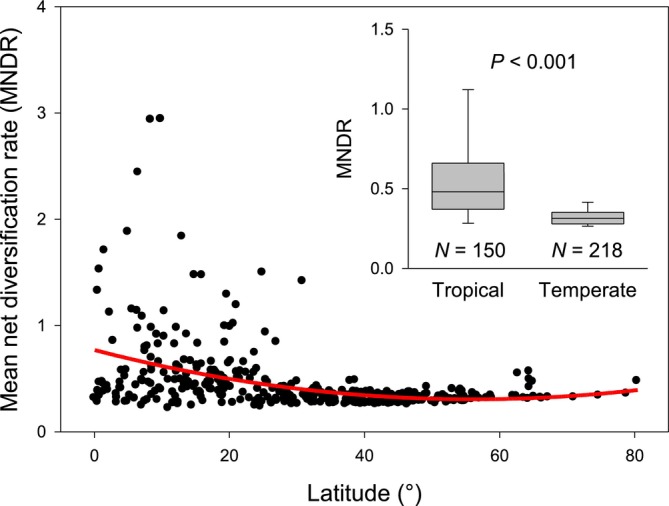
Variation in mean net diversification rate (MNDR) of angiosperms in geographic units across latitude and between tropical and temperate latitudes (inset). The red line is the second‐order polynomial fit to the data points. Inset: A geographic unit is considered as being tropical if its centroid is located between 23.5°N and 23.5°S latitude; otherwise, it is considered as being temperate (either north of 23.5°N or south of 23.5°S latitude). The boxes represent the median and 25th and 75th percentiles, and whiskers the 5th and 95th percentiles. *p* values resulted from *t*‐test of the mean values of mean diversification rates between tropical and temperate geographic units.

We found that using the method‐of‐moments estimator to estimate diversification rate, the mean net diversification rate of angiosperms tends to decrease with latitude (Spearman's rank correlation *ρ* = −0.458; Figure 1). When the geographic units across the world are divided into tropical (between 23.5°N and 23.5°S latitude) and temperate (either north of 23.5°N or south of 23.5°S latitude) groups, the mean net diversification rates of angiosperms are on average almost twice as high in tropical as in temperate latitudes (0.620 vs. 0.341, respectively, *t*‐test, *p* < 0.001; Figure 1). Because the trend of decreasing mean net diversification rates of angiosperms toward high latitudes and the greater average of mean net diversification rates in tropical latitudes than in temperate latitudes were not derived from statistical inference, they are not affected by spatial autocorrelation among geographic units.

## 4 Discussion

The latitudinal gradient of diversification rates in angiosperms that we observed is opposite to that reported in Dimitrov et al. (2023), Igea and Tanentzap (2020), Sun et al. (2020), and Tietje et al. (2022) for angiosperms. Because the present study and Dimitrov et al. (2023) used similar data—the two studies used the same data of genus divergence times, included nearly all of angiosperm species, and used a similar set of geographic units—the differences in the latitudinal pattern of angiosperm diversification rates are likely caused by the use of different diversification rate estimators in the two studies (i.e., BAMM versus the method‐of‐moments estimator).

There is a long debate on whether the method‐of‐moments estimator is more robust than the BAMM estimator. For example, Moore et al. (2016) published an article entitled “Critically evaluating the theory and performance of Bayesian analysis of macroevolutionary mixtures” to criticize BAMM, and Meyer et al. (2018) pointed out in the title of an article that “BAMM gives misleading rate estimates in simulated and empirical datasets”. Meyer and Wiens (2018) pointed out in the title of another article that “Estimating diversification rates for higher taxa: BAMM can give problematic estimates of rates and rate shifts”. In response, to defend BAMM, Rabosky et al. (2017) published an article entitled “Is BAMM flawed? Theoretical and practical concerns in the analysis of multi‐rate diversification models” and another article entitled “BAMM at the court of false equivalency: a response to Meyer and Wiens.” Subsequently, Wiens and colleagues responded to comments from Rabosky and colleagues by stating that Rabosky and colleagues did not address some of the key issues raised by Wiens and colleagues (Wiens 2024; Wu and Wiens 2022; Yu and Wiens 2024). We are not in a position to judge whether the method‐of‐moments estimator is more robust than BAMM, and vice versa. However, the fact that these two methods can result in opposite results for the same or similar data indicates that caution must be exercised in interpreting results from either method. Considering that well resolved species‐level phylogenies remain lacking for most groups of organisms and thus phylogeny‐based diversification estimators cannot be used for them, we predict that the method‐of‐moments estimator will remain a main tool to estimate diversification rates in coming years.

Wiens (2024) points out that no evidence has been provided that the method‐of‐moments estimator performs any worse than other methods, that simulations show that the method‐of‐moments estimator can estimate rates that are strongly correlated with true rates, and that empirical analyses show that diversification rates estimated by the method‐of‐moments estimator are strongly correlated with those from other methods. Thus, we expect that the method‐of‐moments estimator will remain to be a key estimator of diversification rates in future studies, particularly when well‐resolved phylogenies at the species level are not available for study organisms, while acknowledging that using the method‐of‐moments estimator to estimate diversification rates at the genus level, as well as at any other taxonomic levels, may not be able to determine within‐genus evolutionary variation.

The opposite latitudinal gradients of diversification rates observed in Dimitrov et al. (2023) and the present study may not fully be attributed to differences between the method‐of‐moments estimator and BAMM. Dimitrov et al. (2023) did not use BAMM correctly. As we pointed out above, the robustness of BAMM largely depends on the robustness of the phylogeny used with BAMM. The goal of Dimitrov et al.'s study was to determine tip diversification rates of angiosperms (i.e., diversification rates within genera). However, the phylogeny used in their study resolved only at the genus and higher levels, and lacks phylogenetic relationships among species within genera. Because BAMM is not designed for genus‐level phylogenies like the one used by Dimitrov et al., but rather for species‐level phylogenies, using the genus‐level phylogeny to estimate species‐level diversification rates based on BAMM is problematic.

Igea and Tanentzap (2020) and Sun et al. (2020) used BAMM, among other diversification estimators, to estimate angiosperm diversification rates and found that angiosperms diversify more slowly close to the tropics. However, because Igea and Tanentzap (2020) included less than 20% of the global angiosperm species, the result of their study cannot be compared with that of our study, which included nearly all angiosperm species in the world. Furthermore, whether the subset of angiosperm species used in their study is biased toward certain lineages or either climatic zone (i.e., tropical versus temperate) has not been assessed. The result of Sun et al. (2020) also cannot be compared with that of our study partly because their study included only 6% of global angiosperm species and partly because the species examined in their study belong to a single lineage (rosids) of angiosperms. It is well known that diversification rates vary greatly among different lineages (Laenen et al. 2014). Tietje et al. (2022) took a phylogeny‐based metric, which is mean root distance, to estimate diversification rates in global seed plants. Because Tietje et al. (2022) study is based on the phylogeny of seed plants generated by Smith and Brown (2018), in which only about 20% of global seed plant species were resolved, whether the diversification rates estimated in their study are robust remains to be tested. Nevertheless, their finding cannot be directly compared with ours because the two studies covered different taxonomic scopes: Their study included both gymnosperms (which are primarily subtropical and temperate in distribution, and poorly represented in the tropics) and angiosperms whereas ours included only angiosperms.

In sum, on the one hand, the studies by Dimitrov et al. (2023) and Tietje et al. (2022), which included nearly all angiosperm species worldwide, were based on diversification rates derived from phylogeny‐based approaches. Because the vast majority of the species in their studies lack evolutionary histories in their phylogenies, it is difficult to judge how robust the diversification rates estimated in their studies are. On the other hand, the studies by Igea and Tanentzap (2020) and Sun et al. (2020) included less than 20% of global angiosperm species, and whether the findings of their studies based on such a small proportion of global angiosperm species can represent the overall angiosperm flora remains unknown. More data from poorly studied angiosperm groups are needed so that we can move toward developing well‐resolved species‐level phylogenies for angiosperms, which will allow a better assessment of the robustness of various diversification methods.

We thus propose that it is premature to conclude that diversification rates estimated by phylogeny‐based methods such as BAMM and MRD support the notion that diversification rates of angiosperms are higher towards high latitudes due to various problems with previous phylogeny‐based studies on diversification rates of angiosperms, as noted above. Our study based on diversification rates within genera estimated by the method‐of‐moments estimator shows that diversification rates of angiosperms are lower toward high latitudes.

## Author Contributions

**Hong Qian:** conceptualization (lead), investigation (lead), writing – original draft (lead), writing – review and editing (equal). **Michael Kessler:** writing – original draft (equal), writing – review and editing (equal).

## Conflicts of Interest

The authors declare no conflicts of interest.

## Data Availability

No datasets were generated for this manuscript. Data of genus ages are available from Dimitrov et al. (2023). Distributional data of plants were obtained from the World Checklist of Vascular Plants (WCVP) database (https://powo.science.kew.org/about‐wcvp; also available from the website at https://powo.science.kew.org).
